# Ribonuclease E Modulation of the Bacterial SOS Response

**DOI:** 10.1371/journal.pone.0038426

**Published:** 2012-06-08

**Authors:** Robert Manasherob, Christine Miller, Kwang-sun Kim, Stanley N. Cohen

**Affiliations:** 1 Department of Genetics, Stanford University School of Medicine, Stanford, California, United States of America; 2 Department of Medicine, Stanford University School of Medicine, Stanford, California, United States of America; 3 Systems and Synthetic Biology Research Center, Korea Research Institute of Bioscience and Biotechnology (KRIBB), Yuseong-gu, Daejeon, Republic of Korea; Baylor College of Medicine, United States of America

## Abstract

Plants, animals, bacteria, and *Archaea* all have evolved mechanisms to cope with environmental or cellular stress. Bacterial cells respond to the stress of DNA damage by activation of the SOS response, the canonical RecA/LexA-dependent signal transduction pathway that transcriptionally derepresses a multiplicity of genes–leading to transient arrest of cell division and initiation of DNA repair. Here we report the previously unsuspected role of *E. coli* endoribonuclease RNase E in regulation of the SOS response. We show that RNase E deletion or inactivation of temperature-sensitive RNase E protein precludes normal initiation of SOS. The ability of RNase E to regulate SOS is dynamic, as down regulation of RNase E following DNA damage by mitomycin C resulted in SOS termination and restoration of RNase E function leads to resumption of a previously aborted response. Overexpression of the RraA protein, which binds to the C-terminal region of RNase E and modulates the actions of degradosomes, recapitulated the effects of RNase E deficiency. Possible mechanisms for RNase E effects on SOS are discussed.

## Introduction

An ability to maintain genome integrity when threatened by adverse events occurring in the intracellular or extracellular environment is a biologically important trait that has been conserved among plants, animals, bacteria, and *Archaea* [1], [2]. Bacteria commonly react to the specific threat of DNA damage by mounting the SOS response, which assists the restoration of genome integrity and allows survival of DNA-damaged cells [3]. In *E. coli*, the SOS response can be induced by internal events that include stalled replication forks, defective DNA recombination or chromosome segregation, and DNA damage by cell metabolites; extracellular stresses that damage DNA include UV irradiation and noxious chemicals [4].

Initiation of SOS is mediated by a conformational change in the RecA protein upon binding to single stranded segments of damaged DNA [2], [5]. The conformationally altered RecA protein interacts with the transcriptional repressor protein LexA, stimulating LexA self cleavage; this event causes LexA to dissociate from its DNA binding sites, turning on a group of genes (the SOS regulon) whose promoters contain a characteristic sequence (*i.e.*, the “SOS box”) and whose actions lead to the arrest of cell division and facilitate DNA repair [6]. The cessation of cell division and other events that are part of the SOS response help to mitigate or circumvent the otherwise lethal effects of DNA damage seen among actively dividing cells [7]; however, the arrest of cell division is transient. A prolonged SOS response may have a fitness cost [8] and the products of several genes are known to suppress SOS during normal cell growth and/or to have a role in its termination [9]. These SOS-suppressing genes (SSGs) act largely by interfering with the conformation or actions of RecA-DNA filaments [9].

The ribonuclease E (RNase E) family of endoribonucleases–discovered initially in *E. coli*–controls global mRNA degradation as well as the maturation of functional rRNAs, tRNAs, and small regulatory RNAs (for a review, see [10]). Orthologs of RNase E have been identified in more than 50 bacterial species, as well as in *Archaea* and plants [11]. *E. coli* RNase E is a 1061 amino acid single-strand specific endoribonuclease containing three functionally distinct regions: an N-terminal region (amino acid residues 1–529) that includes a catalytically active site [12], [13], an arginine-rich central region, which has a strong RNA-binding ability [13], [14], and a C-terminal region that provides a docking site for multiple proteins (polynucleotide phosphorylase, RhlB helicase, and enolase), which, together with RNase E, form a complex termed the ‘degradosome’ [15], [16]. Recent evidence indicates that degradosome composition, and consequently the cellular actions of RNase E, can be regulated *in vivo* by two ribonuclease E-binding proteins, RraA and RraB as well as by other proteins that interact with the C-terminal region [17], [18], [19]. In *E. coli,* RNase E is normally essential for cell growth; however, the loss of colony forming ability of *rne*-deficient bacteria can be prevented by elevated expression of wild type or mutant forms of the RNase E paralog, ribonuclease G, to twice the normal cellular concentration of RNase E (*i.e.*, 50 times the normal RNase G level) [20], [21].

During studies of biological effects of RNase E on cellular physiology, we observed that cells deficient in RNase E cannot mount or maintain a normal SOS response. This observation led to the investigations reported here, establishing that RNase E can dynamically regulate the SOS response in *E. coli*.

## Results

### Effects of RNase E Deficiency on SOS

**Figure 1:**
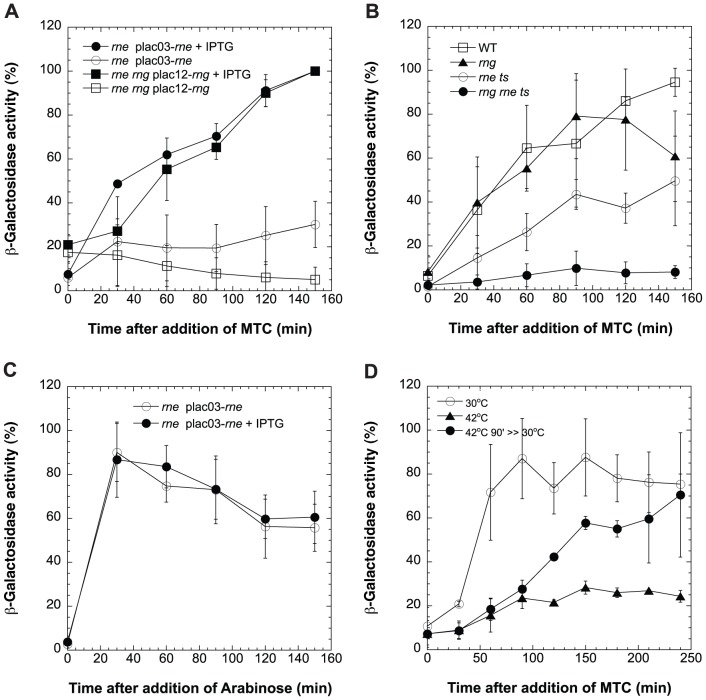
Effect of RNase E and/or G on SOS Response. (A) Effect of *rne* and *rne rng* double null mutations on SOS. β-galactosidase activity encoded by a chromosomally inserted *sulA-lacZ* fusion was measured in *E. coli* cells SC5074 (*rne,* plac03-*rne*, circles) and SC5070 (*rne rng,* plac12-*rng*, rectangles). Cells expressing RNase E or G from an IPTG regulated promoter were cultured, collected, and washed at OD_600_ = 0.1 and then re-cultured for 2 h with or without IPTG to complement or deplete RNase E or G; at time 0 the O.D of the various cultures was adjusted to the same level and SOS was induced by addition of 0.1 µg/ml of MTC. β-galactosidase activity was measured in SC5083 *E. coli* cells grown in the presence of 0.042 mM IPTG to induce RNase E (•) or in cultures lacking IPTG (○). Both RNase E and G were depleted in *E. coli* cells containing an *rne* null mutation complemented by an IPTG-inducible RNase G gene (SC5070; *rne::cm, rng::km*, plac12-*rng*) and then tested for later growth in the presence (▪) or absence (□) of IPTG (0.42 mM). The values are averages of at least three independent experiments calculated as the percent of the highest level of β-galactosidase activity (in Miller Units) accumulated in each experiment and standard deviation is indicated by error bars (bars = s.d.). After completion of the experiments we found that *rng* on the plasmid plac12-*rng* that was overexpressed is a variant containing a point mutation that not interfere with its ability to compliment the *rne*. (B) β-galactosidase activity encoded by a chromosomally inserted *sulA-lacZ* fusion was measured in syngenic strains after addition of MTC (0.1 µg/ml): WT parental cells (SC5080), (□); *rng* deletion, SC5077 (▴); *rne^ts^*, SC5079 (○), double mutant *rng, rne^ts^*, SC5078 (•) (see Table S1). All strains were shifted from 30°C to 42°C at OD_600_ = 0.1, 2 h prior to SOS induction to inactivate the RNase E in *ts* mutants. (C) *De novo* production of β-galactosidase protein from pCM400, Tc^R^ plasmid by *rne* mutant (SC5083, *rne::cm*, plac03-*rne*, pCM400, Tc^R^) cells depleted of RNase E by removal of IPTG, as described for Figure 1A. Arabinose-induced β*-*galactosidase activity was measured after depletion (○), or in the presence of IPTG-induced RNase E (•). (D) Recovery of SOS response after return of SC5078 strain (double mutant *rng, rne^ts^*) to permissive temperature. MTC (0.1 µg/ml) was added at time 0 to cells grown at 30°C for 90 min; β-galactosidase activity was measured in cultures maintained at 30°C (○), shifted to and maintained at 42°C (▴), or returned to 30°C after 90 min incubation at 42°C (•), (bars = s.d.).

To quantitatively evaluate the effects of RNase E activity on SOS, we used a fusion of the *lacZ* reporter gene to the transcriptional control region of the SOS box gene *sulA* (*sfiA),* whose expression is known to be correlated with the SOS event (*cf*., [22]). This reporter construct was introduced into a strain carrying a chromosomal *rne* insertion mutation that was complemented to viability (*i.e.*, to colony forming ability; CFA) by expression of a plasmid-borne gene producing either full length RNase E or RNase G ([20], [21], also see Figure 1 legend) under control of a IPTG-regulated *lac* promoter.

As seen in Figure 1A, RNase E-depleted bacteria showed dramatically reduced *sulA-*mediated *lacZ* expression following treatment by the DNA-damaging agent mitomycin C (MTC). Similarly, transfer of an *rne^ts^* mutant strain (Table S1) to a temperature non-permissive for RNase E function prior to exposure to MTC reduced both the rate and extent of LacZ production from the *sulA-lacZ* fusion (Figure 1B). The effects of absence of RNase E activity on induction of SOS was not attributable to any loss of ability of RNase E-deficient bacteria for the *de novo* synthesis of RNA or protein, as we observed no effect of RNase E absence on induction of β-galactosidase synthesis from an SOS-independent arabinose-controlled promoter (Figure 1C and Figure S2). Bacteria lacking RNase E also continued to incorporate ^3^H-labeled uracil into RNA during the period monitored for effects of RNase E deficiency on SOS, and previously were found to also incorporate a mix of ^3^H-labeled amino acids into protein during the same period of time ([23], and data not shown).

Insertional mutation of *rng* (see Materials and Methods), which encodes the RNase E-related enzyme, RNase G had no detectable effect on induction of SOS in *rne^+^* bacteria (Figure 1B, filled triangles). However, such mutation increased the effects on *sulA-lacZ* production of loss of *rne* activity by either turn off of plasmid-borne *rne* expression in cells chromosomally deleted for the *rne* gene (Figure 1A) or temperature inactivation of RNase E produced by an *rne^ts^* mutant (Figure 1B). Conversely, overexpression of RNase G, which in previous studies has been shown to impart colony-forming ability (CFA) on cells lacking RNase E [20], [21] mitigated the effects of loss of RNase E activity on the SOS response (Figure 1A, open vs. filled squares). The ability of overexpressed RNase G to complement and allow normal SOS response in *rne* mutant cells argues that degradosome formation is not necessary for *E. coli* cells to mount an SOS response, as RNase G lacks the RNase E scaffold necessary for degradosome formation [10], [24].

The above results indicate that RNase E function is required for *E. coli* cells to mount a normal SOS response, that RNase G deficiency enhances the effects of absence of RNase E, and that RNase E-deficient cells remain able to produce RNA and protein during the period when SOS response was inhibited by lack of the enzyme. Evidence that the events that normally would cause cells to mount an SOS response in the presence of RNase E lead to initiation of the SOS when RNase E activity is restored was obtained in an experiment in which doubly mutant *rne^ts^ rng^null^* bacteria that had been treated with MTC at 42°C for 90 min were transferred to 30°C and followed for induction of SOS. As seen in Figure 1D, the expression of *sifA-lacZ*, which had been held in abeyance during RNase E deficiency began to rise following shift of cultures to a temperature that restored function to mutant RNase E protein.

### Effect of the RraA Protein on SOS

**Figure 2:**
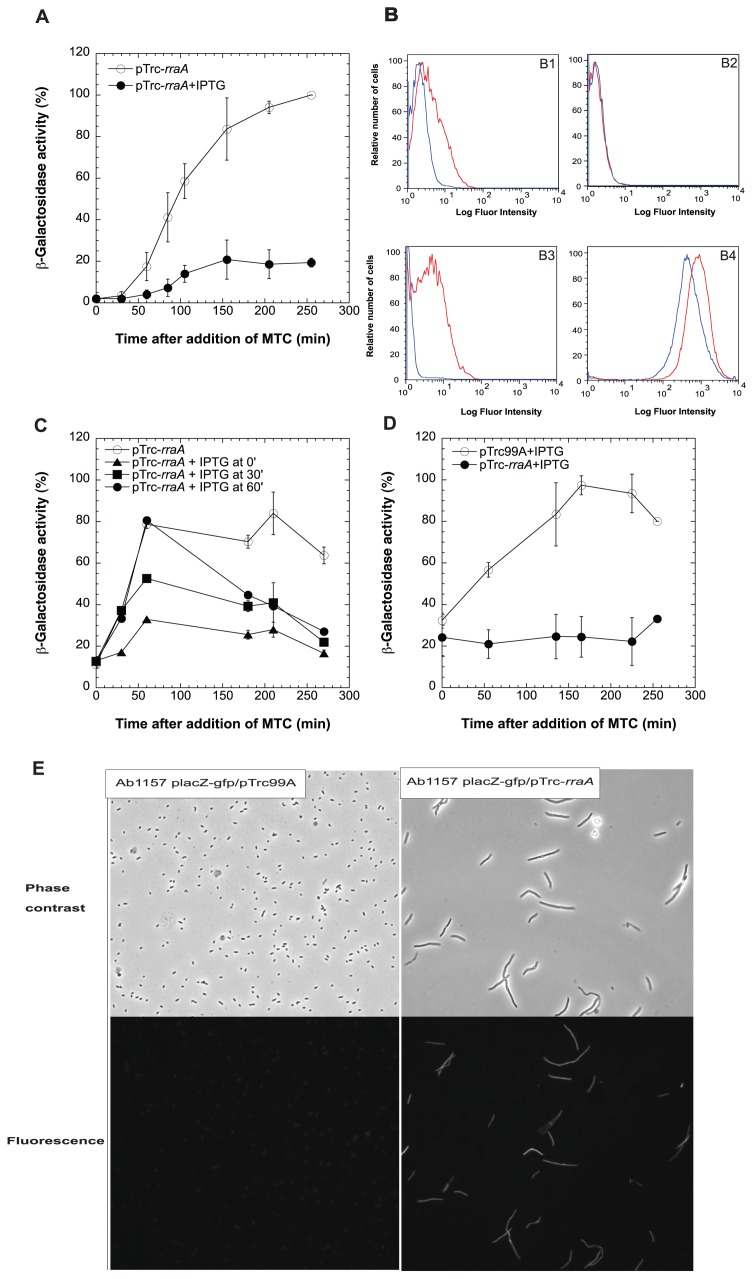
Suppression of SOS response by RraA. (A) Effect of plasmid-borne RraA uninduced (○) or induced with IPTG (•), on chromosomally inserted *sulA-lacZ* fusion expression in SY2 cells. IPTG (1 mM) was added 15 min prior to induction of SOS response at time 0 by MTC (5 µg/ml) and the highest Miller Unit observed in control cells was 2115 (bars = s.d.). (B) FACS quantification of SOS response in individual bacterial cells of SMR6669 (*sulA-GFP)* (Table S1). (B1) SMR6669 cells transformed with pTrc-*rraA* induced with 1 mM IPTG (blue) or uninduced (red). At time 0, SOS response was induced by addition of 1 µg/ml of MTC, and after 90 min cells harvested, washed, re-suspended in PBS and their GFP fluorescence was analyzed by FACScan. (B2) Propidium Iodide (PI) staining was used as functional permeability control to show that the RraA over expressing cells are alive and intact. (B3) SMR6669 cells transformed with pASKA-*rraA* (behind a stronger promoter) induced with IPTG 1 mM (blue) or uninduced (red) at time 0, cells harvested at 90 min after SOS induction by 1 µg/ml of MTC. (B4) SY2 cells harboring plasmid p*lexA*-GFP, a SOS reporter fusion, with pTrc-*rraA* (blue) or pTrc99A empty plasmid (red), both induced by 1 mM IPTG at time 0, cells harvested at 90 min after SOS induction by 1 µg/ml of MTC. (C) Down regulation of ongoing SOS by RraA. After induction of SOS response at time 0 by addition of MTC (0. 1 µg/ml) RraA was induced by IPTG at the indicated times at: 0 min (▴), 30 min (▪), or 60 min (•), (bars = s.d.). (D) Down regulation of spontaneous SOS response by RraA. Basal level of SOS response was measured in SY2 cells containing control plasmid pTrc99A (○) or pTrc-*rraA* (•) after addition of IPTG 1 mM and the highest Miller Unit observed in control cells was 114 (bars = s.d.). (E) Phase contrast and fluorescence images of Ab1157 cells carry placZ-GFP plasmid and pTrc99A or pTrc-rraA plasmid 2 h after induction of RraA and lacZ-GFP with IPTG. Images were taken by Leica DM 5500B microscope.

RraA (*r*egulator of *r*ibonuclease *a*ctivity *A*) is a 17.4 kDa protein that is known to down-regulate the endoribonucleolytic activity of RNase E [17], [18], [19], [24]. As seen in Figure 2A, overexpression of RraA resulted in a decrease in MTC-induced (Figure 2A) or UV irradiation-induced (data not shown) expression of the *sulA-lacZ* reporter. The ability of RraA overexpression to interfere with SOS was additionally demonstrated (Figure 2B1, B3, strain SMR6669) using a chromosomally located green fluorescence protein (*gfp)* reporter gene and fluorescence-activated cell sorting to quantify transcription from the *sulA* promoter in single cells [25], [26]. Further confirmation of the inhibitory effect of RraA overproduction on SOS was obtained by replacing the *sulA* promoter with the promoter regions of other SOS response genes, *lexA* (Figure 2B4) or *dinD* (data not shown) fused to a plasmid-borne *gfp* reporter gene. These results indicate that the observed effects are not unique to *sulA*. As was observed for RNase E, regulation of the SOS response by RraA was dynamic: RraA over production not only prevented the onset of SOS induced by DNA damage, but also aborted an SOS response that had begun 30 or 60 min previously and was still in progress (Figure 2C).

**Figure 3:**
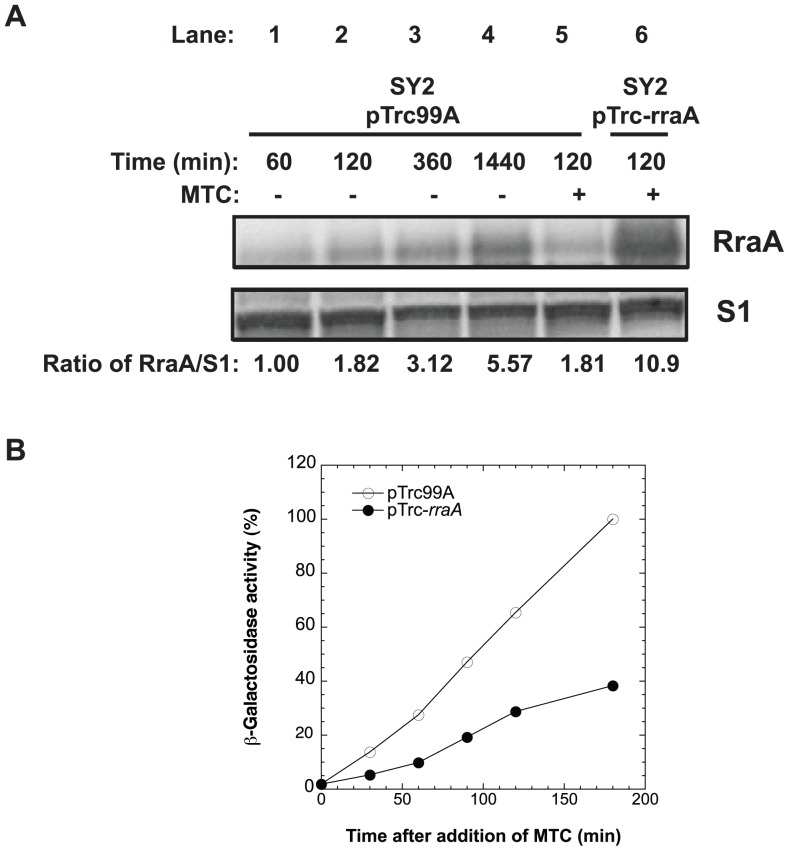
Cell growth effects of RraA-mediated inhibition of SOS. Physiological production of RraA at the level required for down-regulation of SOS. In stationary phase, RraA is produced at levels that can interfere with SOS response induction, demonstrated by western blot (A) and β-galactosidase activity (B). (A) A small amount of RraA protein expressed from the *lacZ* promoter of the pTrc-*rraA* plasmid was sufficient to suppress the SOS response; the level mediating such suppression was compared to the level of RraA protein expressed in stationary phase (after 24 h growth) by untreated SY2 cells harboring pTrc-99A (empty plasmid). Aliquots were assayed by Western blot at various times with and without MTC addition (samples were separated by Criterion 12% Bis-Tris gel electrophoresis using XT-MOPS buffer and were immunoblotted using anti-RraA and anti-S1 antibodies. Specific protein bands were imaged by VersaDoc 1000 (Bio-Rad) and quantified by Quantity One (Bio-Rad). (B) Repression of SOS response by leaky expression of RraA from pTrc-*rraA* plasmid in SY2 cells cultured in the absence of IPTG. SOS response was induced at 0 time in SY2 cells harboring pTrc99A (○, empty plasmid) or pTrc-*rraA* (•) by addition of MTC (0.1 µg/ml).

Not surprisingly, adventitious overexpression of RraA from an IPTG-induced promoter also blocked (Figure 2D) the low-level spontaneous activation of SOS that occurs in small fraction of the population during normal growth of *E. coli* cells as a result of failed replication forks, double-strand DNA breakage, pH changes occurring during the growth cycle, the production of reactive oxygen species or other cell-toxic-products and events associated with the entry of cells into stationary phase [27], [28]. As was observed for cells lacking RNase E function, cells that greatly overexpressed RraA showed defective cell division and commonly form filaments consisting of multiple linked cells (Figure 2E). However, cells that overexpress RraA can produce protein *de novo*, as demonstrated by the production of GFP from a *lacZ-gfp* fusion construct (Figure 2E). We also observed that RraA production occurred from the leaky *lacuv5* promoter on the multicopy pTrc plasmid (Figure 3A, compare lane 5 and 6), and that even this amount of RraA was sufficient to interfere with MTC activation of SOS (Figure 3B, detected at the same time point of 120 min after MTC addition).

RraA expression has been reported to vary during the growth cycle [29], and using antibodies raised against a synthetic RraA peptide, we found that RraA expression increased more than five fold during late log phase growth (Figure 3A; *cf.*, lanes 1 and 4). The cellular abundance of RraA as cells enter stationary phase was comparable to the abundance of RraA protein produced by the uninduced multi-copy plasmid pTrc-*rraA* under *lacuv5* promoter control logarithmically growing cells (Figure 3A; lanes 4 vs. 6), suggesting that SOS induction occurring during normal cell growth may partially be held in check by a concurrent rise in RraA production.

**Figure 4:**
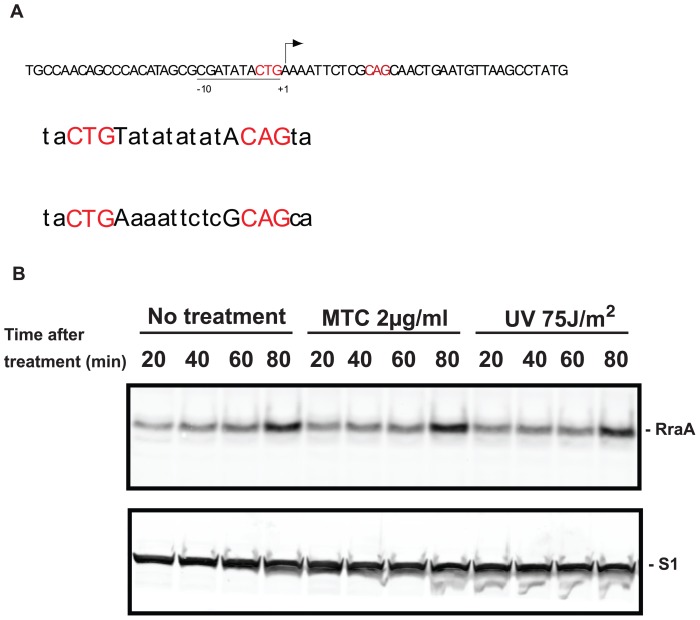
*rraA* contains putative SOS box unaffected by treatment with MTC or exposure to UV light. (A) Putative *rraA* SOS box. (*Top*) Sequence of *rraA* promoter region showing putative SOS box sequence in red. (*Middle*) Proposed [6] consensus SOS box. (*Bottom*) Observed SOS box sequences in *rraA* promoter region. (B) Determination of RraA protein in exponential phase of treated and control SY2 cells to cells after treatment with MTC (2 mg/ml) or exposure to UV light. Aliquots were assayed by Western blotting at various time points and samples were separated by Criterion 12% Bis-Tris gel containing XT-MOPS buffer and immunoblotted using antibodies against RraA. Antibodies to ribosomal protein S1 were used to detect the internal loading control.

Analysis of the promoter region of the *rraA* sequence shows that it includes an *E. coli* consensus SOS box, (Figure 4A); however, we did not detect any increase in RraA protein after induction of SOS response with MTC or UV (Figure 4B).

During these experiments we observed that RraA overexpression induced by IPTG also inhibited the SOS response bacteria that carry chromosomal mutations in *rne* and *rng* and are complemented to viability by overexpression of plasmid-encoded *rng* gene (Figure S1). This result suggests that RraA has additional effects on SOS that are RNase E independent, that RraA can regulate RNase G activity under the conditions tested, or both. The mechanisms underlying such RNase E independent effects of RraA overproduction are under investigation.

## Discussion

The results reported here identify a previously unsuspected role for RNase E and for the ribonuclease regulatory protein RraA, in control of the SOS response. They show that either deficiency of active RNase E or adventitious expression of RraA severely limits both the induction and maintenance of the SOS response, as assessed using a *lacZ* or *gfp* reporter gene linked to promoters of the SOS regulon genes (principally *sulA*, but also *lexA* and *dinD*). Mutation of the RNase E paralog, RNase G, enhanced the effects of RNase E deficiency, and overexpression of RNase G (and see Figure 1 legend), which allows complementation of RNase E deficiency enabled SOS induction to occur. Thus, RNase G overexpression can mitigate the effects of RNase E limitation on SOS induction. However, RNase G was itself not required for SOS.

Activation of more than 30 genes occurs during SOS induction, and the *de novo* production of proteins encoded by many of these genes has been shown to be necessary for a normal SOS response [30]. Our data show that RNase E deficient cells continue to produce proteins during the time of monitoring the effects of the enzyme on SOS. These findings confirm earlier observations made by Goldblum and Apririon, showing that RNA, DNA and protein synthesis continue for at least 3 h following the shift of an *rne^ts^* mutant to a non-permissive temperature [23]. Additionally, expression of SOS regulon genes in an *rne^ts^* strain cultured at a non-permissive temperature was initiated upon subsequent shift to a temperature that restored RNase E function, indicating that the effects of RNase E deficiency on SOS are reversible, and thus that SOS control by RNase E is a dynamic event. Cells that over-express RraA also were able to produce LacZ –GFP fused proteins *de novo* during the period that SOS was inhibited, indicating that in this case also the abrogation of SOS is not attributable to any global defect in protein synthesis. The reduction of SOS response in cells over expressing RraA is not the result of any toxic effects of the over expression *per se,* as cells stained with propidium iodide (PI), a functional permeability control, confirmed that the RraA overexpressing cells were alive and their membranes were intact (Figure 2B2) during the period monitored for SOS response reduction.

The effect of RraA on the SOS response was additionally confirmed and quantified by SOS expression in individual bacterial cells, which was tracked via a chromosomally located green fluorescent protein (GFP) gene controlled by the SOS-inducible *sulA* promoter [25], [26]. It was shown previously in this system (SMR6669 cells), that green fluorescence resulting from SOS induction allows sensitive detection by flow cytometry of events that affect SOS [25], [26]. Adventitious expression of RraA sharply diminished fluorescence produced by expression of the *sulA*-GFP fusion following treatment by MTC (Figure 2B1, B3), and was shown to also down-regulate promoters of the SOS response genes *lexA* and *dinD*.

Zhao *et al.* have reported doubling of the steady state level of RraA protein during the stationary phase of the *E. coli* growth cycle [29]. We confirmed the effects of the growth cycle on RraA abundance, and using antibodies raised against a synthetic RraA peptide, found that RraA expression increased during late log phase growth. The increased RraA abundance observed in logarithmically growing cells entering stationary phase [more than five fold (Figure 3A, lane 4)] is comparable to the RraA abundance observed in cells undergoing suppression of MTC-induced SOS by an uninduced *rraA* gene carried by a multicopy plasmid (Figure 3B and Figure 3A, lane 6). This finding argues that normally occurring physiological levels of RraA are sufficient to modulate the SOS response, suggesting that differences in RraA abundance occurring at different times during the bacterial growth cycle, or perhaps during cell growth under different environmental conditions, may alter the bacterial response to DNA damage. The findings further suggest that the up-regulation of RraA observed as cells approach stationary phase may limit the SOS induction that occurs as normal cells proceed through the growth cycle.

Analysis of the promoter region of the *rraA* gene sequence shows that it includes an *E. coli* consensus SOS box, a characteristic of LexA-regulated genes (Figure 4A). While this finding raised the prospect that that RraA may itself be an SOS induced gene that regulates SOS by a feedback control mechanism, we observed no increase in RraA protein after induction of SOS response with MTC or UV (Figure 4B) indicating that the putative SOS box in the RraA promoter region does not respond to *lexA-*modulated derepression under the conditions tested. However, LexA has been reported to bind to sequences having a ‘Heterology Index’ (HI) value of 12.6 or lower [31], [32]. We calculate the HI index of *rraA* SOS box and found that it was 11.6. Collectively, these findings raise that prospect that LexA regulation of *rraA* expression may occur under still-unidentified conditions.

RNase E has global role in RNA turnover, and included among the transcripts whose cellular abundance is affected by RNase E mutation [33] are those encoding genes known to suppress or otherwise affect the SOS response. It has not been determined whether altered degradation of such transcripts is causally related to the effects of RNase E deficiency on SOS response induction and maintenance.

The ability of cells to adjust protein levels rapidly enhances their chance of survival after severe environmental stress. The DNA-damage response–in which recombination, DNA-repair, lesion-bypass, and cell-cycle checkpoint proteins are induced–is highly conserved among most organisms from bacteria to mammals [34]. In *E. coli*, most cellular proteins have long half-lives [35], [36], but in contrast, many of the ∼40 proteins expressed in a LexA-dependent manner are unusually labile to proteolysis. Nine of these proteins are ClpXP protease substrates based on trapping and/or degradation experiments [37], [38], [39], while at least six DNA-damage induced proteins appear to be Lon substrates [40], [41], [42]. Moreover, among the eight proteins that are most highly induced after DNA damage, seven are rapidly degraded [43]. Highlighting the importance of fast turnover of SOS response proteins, several of them are degraded by multiple proteases. The results reported here add ribonuclease-mediated regulation the previously studied multifaceted aspects of protein control of SOS. To function at a systems level, ribonuclease control of SOS must necessarily be integrated with the other aspects of SOS control, such as the synthesis of SOS suppressor genes, modulation of lexA regulon protein production, and proteolysis. As SOS induction is known to facilitate bacterial resistance to antibiotics [44], [45], [46] as well as bacterial virulence [47] we speculate that downregulation of RNase E or molecules that function similarly to RraA may prove useful as adjuncts to antimicrobial therapy.

## Materials and Methods

### DNA Manipulations and Analysis

Standard procedures were performed as described previously [48]. Extraction of DNA from bacteria was performed using QIAPrep Spin Miniprep Kit (Qiagen, Valencia, CA, USA). Restriction enzymes were used according to the vendor’s directions (New England BioLabs Inc., Ipswich, MA, USA).

### Measurement of β-Galactosidase Activity

β-galactosidase activity in whole cells was determined as described by Miller 1992 [49]. The values reported are averages of at least three independent experiments calculated as a percentage of the highest level of β-galactosidase activity (in Miller Units) accumulated in each experiment, and standard deviation is indicated by error bars.

### Flow Cytometry Analysis

Experiments were done using log-phase cells (SMR6669) harboring pTrc99A, pTrc-*rraA,* pLacZ-GFP *or* pASKA*-rraA* plasmids, cultured by vigorous aeration at 37°C in LB after induction of SOS response by mitomycin C (MTC) or during overexpression of RraA by IPTG. Cells were harvested and washed twice with and re-suspended in PBS. Fluorescence was measured by FACScan (Becton-Dickinson, San Jose, CA, USA) and analyzed by Cell Quest program. Data were collected from 50,000 events for each sample and all analyses were repeated at least twice for each experiment. Data were analyzed using FlowJo software (Tree Star, Ashland, OR, USA). Propidium iodide, which is excluded from cells having intact membranes but stains dead cells undergoing membrane depolarization, was used to determine cell viability [50]. Cells fluorescing red due to PI staining were detected by flow cytometry simultaneously with detection of GFP.

### Immunodetection of RraA

Polyclonal RraA antibodies were obtained from rabbits inoculated with RraA peptide (N’-^aa40^CFEDNGLLYDLLEQNGRGRV^ aa60^) by Proteintech Group, Inc. (Chicago, IL, USA) and were affinity purified. Secondary goat anti-rabbit IgG (Promega, Madison, WI, USA) antibodies conjugated to horseradish peroxidase were used at 1∶5,000 to 1∶15,000 dilutions (Santa Cruz Biotechnology, Santa Cruz, CA, USA). Antibodies detecting the ribosomal protein S1 were used [51] to provide an internal standard to evaluate the amount of cell extracts in different lanes. Cellular proteins were detected by separating them on Criterion XT Bis-Tris gels (Bio-Rad Laboratories, Hercules, CA, USA), followed by Western blotting using antibody against the protein of interest, and then appropriate secondary antibody and ECL detection reagents (EMD Millipore, Billerica, MA, USA). Specific proteins were imaged using a VersaDoc 1000 (Bio-Rad) and quantified by Quantity One (Bio-Rad).

### UV Irradiation

SOS response induction by UV was carried out using a General Electric 15 watt germicidal lamp having a maximum output of 254 nm. Cells were irradiated for 2 min in an open Petri dish placed 25 cm beneath the lamp.

### Incorporation of ^3^H-Labeled Precursors

Parental (MG1693) and doubly mutant (SK2541 *rng::cat, rne^ts^*) cells were grown as in Figure 1, including a 2 h preincubation at the non-permissive temperature. ^3^H-labeled uracil or ^3^H-labeled amino acids mix (Amersham, GE Healthcare Life Sciences, Pittsburgh, PA, USA) was added; cells were taken at the indicated times and added to 10% TCA for precipitation of nuclei acids and proteins as described previously [48].

### Induction of SOS

Mitomycin C (MTC; Sigma-Aldrich, St. Louis, MO, USA) or UV irradiation were used as indicated in Figure legends. We found that different batches of MTC used over an extended period of time had different potency as inducers of SOS, and different concentrations were employed as necessary to ensure a strong SOS response. In all cases, experimental determinations and controls for these determinations were done using the same batch.

### Disruption of Chromosomal Genes

Cells with *rne* and/or *rng* insertion mutations were constructed by phage P1-mediated transduction using N3433*rne* and N3433*rng* as donor strains [20].

## Supporting Information

Figure S1 **Suppression of SOS response by RraA overexpression independently to RNase E activity.** β-galactosidase activity encoded by a chromosomally inserted *sulA-lacZ* fusion was measured in *E. coli* doubly mutant cells (*rne, rng*) complemented by overexpression of RNase G from pBAD-*rng* plasmid (0.1% arabinose) and harboring pTrc99A (▪, RM1001) or pTrc-*rraA* (•, RM1002) plasmids. At zero time MTC (1 µg/ml) and IPTG (0.1 mM) were added to the cultures to induce SOS response and RraA overproduction, respectively (bars = s.d.).
(EPS)

Figure S2 ***De novo*** **production of β-galactosidase protein in presence of MTC and depletion of RNase E.** β-galactosidase production from pCM400, Tc^R^ plasmid by *rne* mutant (SC5083-BB, *rne::cm*, plac03-*rne*, pCM400, Tc^R^) cells depleted of RNase E by removal of IPTG, as described for Fig. 1A. Arabinose-induced β*-*galactosidase activity was measured in the presence of MTC (1 µg/ml) after depletion (○), or in the presence of IPTG-induced RNase E (•). Since the strain used in Figure 1C (SC5083) contains *sulA-lacZ* fusion, the same strain was used, but without the SOS response gene fused to *lacZ* (SC5083-BB), thus it was possible to follow up after induction of *lacZ* in the presence of MTC, as well.
(EPS)

Table S1 **Strains and plasmids used in this study.**
(DOC)

## References

[1] Seitz EM, Brockman JP, Sandler SJ, Clark AJ, Kowalczykowski SC (1998). RadA protein is an archaeal RecA protein homolog that catalyzes DNA strand exchange.. Genes Dev.

[2] Walker GC, Marsh L, Dodson L (1985). Cellular responses to DNA damage.. Environ Health Perspect.

[3] Radman M (1975). SOS repair hypothesis: phenomenology of an inducible DNA repair which is accompanied by mutagenesis.. Basic Life Sci.

[4] Butala M, Zgur-Bertok D, Busby SJ (2009). The bacterial LexA transcriptional repressor.. Cell Mol Life Sci.

[5] Sassanfar M, Roberts JW (1990). Nature of the SOS-inducing signal in *Escherichia coli*. The involvement of DNA replication.. J Mol Biol.

[6] Kenyon CJ, Walker GC (1980). DNA-damaging agents stimulate gene expression at specific loci in *Escherichia coli*.. Proc Natl Acad Sci U S A.

[7] Simmons LA, Foti JJ, Cohen SE, Walker GC, Böck A, III RC, Kaper JB, Karp PD, Neidhardt FC (2008). The SOS Regulatory Network..

[8] McKenzie GJ, Rosenberg SM (2001). Adaptive mutations, mutator DNA polymerases and genetic change strategies of pathogens.. Curr Opin Microbiol.

[9] Cox MM (2007). Regulation of bacterial RecA protein function.. Crit Rev Biochem Mol Biol.

[10] Cohen SN, McDowall KJ (1997). RNase E: still a wonderfully mysterious enzyme.. Mol Microbiol.

[11] Lee K, Cohen SN (2003). A *Streptomyces coelicolor* functional orthologue of *Escherichia coli* RNase E shows shuffling of catalytic and PNPase-binding domains.. Mol Microbiol.

[12] Callaghan AJ, Marcaida MJ, Stead JA, McDowall KJ, Scott WG (2005). Structure of *Escherichia coli* RNase E catalytic domain and implications for RNA turnover.. Nature.

[13] McDowall KJ, Cohen SN (1996). The N-terminal domain of the rne gene product has RNase E activity and is non-overlapping with the arginine-rich RNA-binding site.. J Mol Biol.

[14] Taraseviciene L, Bjork GR, Uhlin BE (1995). Evidence for an RNA binding region in the *Escherichia coli* processing endoribonuclease RNase E. J Biol Chem.

[15] Py B, Higgins CF, Krisch HM, Carpousis AJ (1996). A DEAD-box RNA helicase in the *Escherichia coli* RNA degradosome.. Nature.

[16] Miczak A, Kaberdin VR, Wei CL, Lin-Chao S (1996). Proteins associated with RNase E in a multicomponent ribonucleolytic complex.. Proc Natl Acad Sci U S A.

[17] Gao J, Lee K, Zhao M, Qiu J, Zhan X (2006). Differential modulation of *E. coli* mRNA abundance by inhibitory proteins that alter the composition of the degradosome.. Mol Microbiol.

[18] Gorna MW, Pietras Z, Tsai YC, Callaghan AJ, Hernandez H (2010). The regulatory protein RraA modulates RNA-binding and helicase activities of the *E. coli* RNA degradosome.. RNA.

[19] Lee K, Zhan X, Gao J, Qiu J, Feng Y (2003). RraA. a protein inhibitor of RNase E activity that globally modulates RNA abundance in *E. coli*.. Cell.

[20] Lee K, Bernstein JA, Cohen SN (2002). RNase G complementation of *rne* null mutation identifies functional interrelationships with RNase E in *Escherichia coli*.. Mol Microbiol.

[21] Tamura M, Lee K, Miller CA, Moore CJ, Shirako Y (2006). RNase E maintenance of proper FtsZ/FtsA ratio required for nonfilamentous growth of *Escherichia coli* cells but not for colony-forming ability.. J Bacteriol.

[22] Huisman O, D’Ari R, Gottesman S (1984). Cell-division control in *Escherichia coli*: specific induction of the SOS function SfiA protein is sufficient to block septation.. Proc Natl Acad Sci U S A.

[23] Goldblum K, Apririon D (1981). Inactivation of the ribonucleic acid-processing enzyme ribonuclease E blocks cell division.. J Bacteriol.

[24] Carpousis AJ (2007). The RNA degradosome of *Escherichia coli*: an mRNA-degrading machine assembled on RNase E. Annu Rev Microbiol.

[25] Hastings PJ, Slack A, Petrosino JF, Rosenberg SM (2004). Adaptive amplification and point mutation are independent mechanisms: evidence for various stress-inducible mutation mechanisms.. PLoS Biol.

[26] Pennington JM, Rosenberg SM (2007). Spontaneous DNA breakage in single living *Escherichia coli* cells.. Nat Genet.

[27] Cox MM, Goodman MF, Kreuzer KN, Sherratt DJ, Sandler SJ (2000). The importance of repairing stalled replication forks.. Nature.

[28] Dri AM, Moreau PL (1994). Control of the LexA regulon by pH: evidence for a reversible inactivation of the LexA repressor during the growth cycle of *Escherichia coli*.. Mol Microbiol.

[29] Zhao M, Zhou L, Kawarasaki Y, Georgiou G (2006). Regulation of RraA, a protein inhibitor of RNase E-mediated RNA decay.. J Bacteriol.

[30] Michel B (2005). After 30 years of study, the bacterial SOS response still surprises us.. PLoS Biol.

[31] Berg OG, von Hippel PH (1988). Selection of DNA binding sites by regulatory proteins. II. The binding specificity of cyclic AMP receptor protein to recognition sites.. J Mol Biol.

[32] Lewis LK, Harlow GR, Gregg-Jolly LA, Mount DW (1994). Identification of high affinity binding sites for LexA which define new DNA damage-inducible genes in *Escherichia coli*.. J Mol Biol.

[33] Bernstein JA, Lin PH, Cohen SN, Lin-Chao S (2004). Global analysis of *Escherichia coli* RNA degradosome function using DNA microarrays.. Proc Natl Acad Sci U S A.

[34] Friedberg E, Walker G, Seide W, Wood R, Schultz R (2006). DNA Repair and Mutagenesis.. Washington, DC: ASM Press.

[35] Mosteller RD, Goldstein RV, Nishimoto KR (1980). Metabolism of individual proteins in exponentially growing *Escherichia coli*.. J Biol Chem.

[36] Pine MJ (1970). Steady-state measurement of the turnover of amino acid in the cellular proteins of growing *Escherichia coli*: existence of two kinetically distinct reactions.. J Bacteriol.

[37] Gonzalez M, Rasulova F, Maurizi MR, Woodgate R (2000). Subunit-specific degradation of the UmuD/D’ heterodimer by the ClpXP protease: the role of trans recognition in UmuD’ stability.. EMBO J.

[38] Neher SB, Flynn JM, Sauer RT, Baker TA (2003). Latent ClpX-recognition signals ensure LexA destruction after DNA damage.. Genes Dev.

[39] Neher SB, Sauer RT, Baker TA (2003). Distinct peptide signals in the UmuD and UmuD’ subunits of UmuD/D’ mediate tethering and substrate processing by the ClpXP protease.. Proc Natl Acad Sci U S A.

[40] Mizusawa S, Gottesman S (1983). Protein degradation in *Escherichia coli*: the lon gene controls the stability of *sulA* protein.. Proc Natl Acad Sci U S A.

[41] Frank EG, Ennis DG, Gonzalez M, Levine AS, Woodgate R (1996). Regulation of SOS mutagenesis by proteolysis.. Proc Natl Acad Sci U S A.

[42] Little JW, Friedberg E, Bridges B (1983). Variations in the *in vivo* stability of LexA repressor during the SOS regulatory cycle..

[43] Neher SB, Villen J, Oakes EC, Bakalarski CE, Sauer RT (2006). Proteomic profiling of ClpXP substrates after DNA damage reveals extensive instability within SOS regulon.. Mol Cell.

[44] Cirz RT, Chin JK, Andes DR, de Crecy-Lagard V, Craig WA (2005). Inhibition of mutation and combating the evolution of antibiotic resistance.. PLoS Biol.

[45] Cirz RT, Jones MB, Gingles NA, Minogue TD, Jarrahi B (2007). Complete and SOS-mediated response of *Staphylococcus aureus* to the antibiotic ciprofloxacin.. J Bacteriol.

[46] Miller C, Thomsen LE, Gaggero C, Mosseri R, Ingmer H (2004). SOS response induction by beta-lactams and bacterial defense against antibiotic lethality.. Science.

[47] Maiques E, Ubeda C, Campoy S, Salvador N, Lasa I (2006). beta-lactam antibiotics induce the SOS response and horizontal transfer of virulence factors in *Staphylococcus aureus*.. J Bacteriol.

[48] Sambrook J, Fritsch EF, Maniatis T (1989). Molecular Cloning.. A Laboratory Manual Cold Spring Harbor, NY: Cold Spring Harbor Laboratory.

[49] Miller JH (1992). A short course in bacterial genetics: a laboratory manual and handbook for *Escherichia coli* and related bacteria.. Cold Spring Harbor, NY: Cold Spring Harbor Laboratory.

[50] Nebe-von-Caron G, Stephens PJ, Hewitt CJ, Powell JR, Badley RA (2000). Analysis of bacterial function by multi-colour fluorescence flow cytometry and single cell sorting.. J Microbiol Methods.

[51] Feng Y, Huang H, Liao J, Cohen SN (2001). *Escherichia coli* poly(A)-binding proteins that interact with components of degradosomes or impede RNA decay mediated by polynucleotide phosphorylase and RNase E. J Biol Chem.

