# Supplementary material for: Ribonuclease E Modulation of the Bacterial SOS Response
Source: PLoS One. 2012 Jun 8;7(6):e38426. doi: 10.1371/journal.pone.0038426 (PMC3374874; doi:10.1371/journal.pone.0038426)
Supplement: Table S1 — Strains and plasmids used in this study. (DOC) [file pone.0038426.s003.doc]

**Table S1 Strains and plasmids used in this study.**

| **Strain** | **Description** | **Reference or source** |
| --- | --- | --- |
| MG1693 | *thyA715, rph-1* | [1] |
| SK2538 | *thyA715, rph-1, rng::cat* | [1] |
| SK5665 | *thyA715, rph-1, rne^ts^* | [1] |
| SK2541 | *thyA715, rph-1, rng::cat, rne^ts^* | [1] |
| SY2 | MC1061 *sulA*::*lacZ*9*YA*::*kan* | [2] |
| PN104 | *garB10, fhuA22, lacZ8(am), phoA4(am), ompF627(T2R), fadL701(T2R), relA1, pit-10, dinD1::Mud(Ap^R^,lac), spoT1, pirA1::KmR, rrnB-2, mcrB1, creC510* | [3] |
| BR5171 | MC4100 Sm^r^ *araD139* Δ[*lacIPOZIA*] *U169 strA* *thi* L(*c*I *ind* *sfiA*::*lacZ)* | [4] |
| AG1 | *recA1 endA1 gyrA96 thi-1 hsdR17* (r_K_- m_K_^+^) *supE44 relA1* | [5] |
| SMR6669 | MG1655 Δattλ::P*sulA*Ω*gfp*-*mut*2 | [6] |
| SC5070 | BR5171 *rne*::*cm* *rng*::*kan* plac12-*rng* | This study |
| SC5074 | MG1655 F^-^ λ^-^ *ilvG* *rfb50* *rph*-1 *sulA*::*lacZ*9*YA*::*kan rne*::*cm*  plac03-*rne* | This study |
| SC5074-BB | MG1655 F^-^ λ^-^ *ilvG* *rfb50* *rph*-1 *rne*::*cm*  plac03-*rne* | This study |
| SC5076 | MG1655 *sulA*::*lacZ*9*YA*::*kan rne*::*cm* plac12-*rng* | This study |
| SC5077 | SK2538 *rng::cat, sulA::lacZ::km* | This study |
| SC5078 | SK2541 *rng::cat, rne^ts^, sulA::lacZ::km* | This study |
| SC5079 | SK5665 *rne^ts^, sulA::lacZ::km* | This study |
| SC5080 | MG1693, *sulA::lacZ::km* | This study |
| SC5083 | SC5074 with pCM400 | This study |
| SC5083-BB | SC5074-BB with pCM400 | This study |
| RM1001 | MG1655 *sulA*::*lacZ*9*YA*::*kan rne*::*cm rng*::*km* pBAD-*rng* pTrc99A | This study |
| RM1002 | MG1655 *sulA*::*lacZ*9*YA*::*kan rne*::*cm rng*::*km* pBAD-*rng* pTrc-*rraA* | This study |
| **Plasmid** | **Description** | **Reference or source** |
| plac03-*rne* | Plac with *rne* under *lac* promoter, pSC101 ori, Ap^R^ | [7] |
| plac12-*rng* | Plac with *rng* (from pRNG3, short form of RNase G) under *lac* promoter, pSC101 ori, Ap^R^ | This study |
| pBAD-myc- hisB | P_BAD_lacZ pBR322 ori, Ap^R^ | Invitrogen |
| pCM400 | pBAD-myc-hisB-lacZ but TC^R^ | This study |
| pTrc99A | ColE1 ori, Ap^R^ | [8] |
| pTrc-*rraA* | pTrc99A with *rraA* under *trc* promoter | [8] |
| plexA-GFP | *lex*A promoter fused to GFP | [9] |
| placZ-GFP | *lacZ* promoter fused to GFP | [9] |
| pASKA-*rraA* | From ASKA library | [5] |
| pKR5 | pBAD-*myc*-*hisB*-cloDF13 Sp^R^ | This study |
| pKR5- *rraA* | *rraA* cloned into pKR5 | This study |
| pBAD-*rng* | pSC101 ori, KM^R^ *rng* under an arabinose inducible promoter | [7] |

**References for Table S1**

1. Ow MC, Perwez T, Kushner SR (2003) RNase G of *Escherichia coli* exhibits only limited functional overlap with its essential homologue, RNase E. Mol Microbiol 49: 607-622.

2. Ohmori H, Saito M, Yasuda T, Nagata T, Fujii T, et al. (1995) The pcsA gene is identical to *dinD* in *Escherichia coli*. J Bacteriol 177: 156-165.

3. Nurse P, Zavitz KH, Marians KJ (1991) Inactivation of the *Escherichia coli* priA DNA replication protein induces the SOS response. J Bacteriol 173: 6686-6693.

4. Huisman O, D'Ari R, Gottesman S (1984) Cell-division control in *Escherichia coli*: specific induction of the SOS function SfiA protein is sufficient to block septation. Proc Natl Acad Sci U S A 81: 4490-4494.

5. Kitagawa M, Ara T, Arifuzzaman M, Ioka-Nakamichi T, Inamoto E, et al. (2005) Complete set of ORF clones of *Escherichia coli* ASKA library (a complete set of *E. coli* K-12 ORF archive): unique resources for biological research. DNA Res 12: 291-299.

6. Hastings PJ, Slack A, Petrosino JF, Rosenberg SM (2004) Adaptive amplification and point mutation are independent mechanisms: evidence for various stress-inducible mutation mechanisms. PLoS Biol 2: e399.

7. Lee K, Bernstein JA, Cohen SN (2002) RNase G complementation of *rne* null mutation identifies functional interrelationships with RNase E in *Escherichia coli*. Mol Microbiol 43: 1445-1456.

8. Lee K, Zhan X, Gao J, Qiu J, Feng Y, et al. (2003) RraA. a protein inhibitor of RNase E activity that globally modulates RNA abundance in *E. coli*. Cell 114: 623-634.

9. Friedman N, Vardi S, Ronen M, Alon U, Stavans J (2005) Precise temporal modulation in the response of the SOS DNA repair network in individual bacteria. PLoS Biol 3: e238.
